# TYROBP-positive endothelial cell-derived TWEAK as a promoter of osteosarcoma progression: insights from single-cell omics

**DOI:** 10.3389/fonc.2023.1200203

**Published:** 2023-05-03

**Authors:** Zhi-qiang Wei, Sheng Ding, Yan-cai Yang

**Affiliations:** The Department of Pediatric Surgery, The Ningbo Women and Children’s Hospital, Ningbo, China

**Keywords:** TWEAK, osteosarcoma, TYROBP, tumor microenvironment, prognosis, endothelial cells

## Abstract

**Background:**

Endothelial cells (ECs) play a vital role in promoting the progression of malignant cells, and they exhibit heterogeneity in their phenotypic characteristics. We aimed to explore the initiating cells of ECs in osteosarcoma (OS) and investigate their potential interaction with malignant cells.

**Method:**

We obtained scRNA-seq data from 6 OS patients, and datasets were batch-corrected to minimize variations among samples. Pseudotime analysis was performed to investigate the origin of differentiation of ECs. CellChat was employed to examine the potential communication between endothelial cells and malignant cells, and gene regulatory network analysis was performed to identify transcription factor activity changes during the conversion process. Importantly, we generated TYROBP-positive ECs *in vitro* and investigated its role in OS cell lines. Finally, we explored the prognosis of specific ECs cluster and their impact on the tumor microenvironment (TME) at the bulk transcriptome level.

**Results:**

The results showed that TYROBP-positive ECs may play a crucial role in initiating the differentiation of ECs. TYROBOP-positive endothelial cells (ECs) exhibited the strongest crosstalk with malignant cells, likely mediated by TWEAK, a multifunctional cytokine. TYROBP-positive ECs exhibited significant expression of TME-related genes, unique metabolic and immunological profiles. Importantly, OS patients with low enrichment of TYROBP-positive ECs had better prognoses and a lower risk of metastasis. Finally, vitro assays confirmed that TWEAK was significantly increased in ECs-conditioned medium (ECs-CM) when TYROBP was over-expressed in EC cells, and could promote the proliferation and migration of OS cells.

**Conclusion:**

We concluded that TYROBP-positive ECs may be the initiating cells and play a crucial role in the promotion of malignant cell progression. TYROBP-positive ECs have a unique metabolic and immunological profile and may interact with malignant cells through the secretion of TWEAK.

## Introduction

Osteosarcoma (OS) is a highly malignant bone tumor, mainly affecting children and adolescents (1). With the development of multimodal therapy, including surgery, chemotherapy, and radiation therapy, the survival rate of OS patients has improved significantly over the past few decades (2). Current research efforts on OS treatment are mainly focused on two aspects: identifying novel therapeutic targets and improving the effectiveness of existing therapies (3). Several potential therapeutic targets for OS have been identified, including receptor tyrosine kinases, immune checkpoints, and metabolic pathways (4, 5). Moreover, advances in genomics and transcriptomics have led to the identification of several molecular subtypes of OS, which may have different treatment responses and prognoses (6). Understanding the molecular characteristics of OS can help to develop personalized treatment strategies and improve patient outcomes.

The tumor microenvironment (TME) plays a crucial role in the progression of OS (7). For example, studies have shown that the TME can affect the behavior of OS cells by promoting angiogenesis, remodeling of the extracellular matrix, and immune evasion (8). Additionally, studies have shown that the TME can influence the response of OS to chemotherapy and radiation therapy (9). Endothelial cells (ECs), the major component of vascular endothelium, have important impacts on tumor growth and metastasis in cancer (10). In cancer, ECs can be classified into multiple subtypes with different transcriptional profiles, phenotypes, and functions (7). The expression profiles and distribution of endothelial cell subtypes are closely associated with different tumor types and microenvironments. Specific proteins expressed by ECs in tumor vasculature can be used for screening potential therapeutic targets, and different subtypes of ECs may respond differently to therapeutic drugs (7). In some cases, ECs can suppress tumor immune responses by expressing immune checkpoint molecules such as PD-L1 (11), and some studies have suggested that tumor vasculature ECs can promote tumor immune evasion and metastasis (12). Therefore, a deeper understanding of endothelial cell heterogeneity in cancer can not only promote the understanding of the tumor microenvironment but also provide valuable insights for the development of novel cancer therapeutic strategies.

The impact of single-cell omics methods on OS and other tumors in the introduction. Indeed, single-cell omics methods have revolutionized our ability to study tumors at the single-cell level, providing unprecedented insights into tumor heterogeneity, cell-to-cell communication, and cellular plasticity (13). Single-cell RNA sequencing (scRNA-seq) is particularly powerful, allowing us to identify rare cell populations and study their transcriptional profiles in depth. In this study, we analyzed scRNA-seq data from OS patients and identified heterogeneity of ECs across different patients. Specifically, we observed that TYROBP-positive ECs showed the strongest interaction with malignant cells and may serve as initiating cells by secreting TWEAK and other proteins that influence tumor progression. We also found that patients with low enrichment of TYROBP-positive ECs had better prognoses and a lower risk of metastasis.

## Materials and methods

### Pre-processing of the scRNA-seq datasets

We obtained the scRNA-seq dataset for osteosarcoma comprising of 6 samples (GSE162454) and the corresponding meta information from the Tumor Immune Single-cell Hub (TISCH) database (14). To ensure the quality of the data, we conducted quality control (QC), normalization, scaling, and principal component analysis (PCA) using the Seurat package (v4.0.5). We then performed clustering using the “Find Neighbors” and “Find Clusters” functions at a resolution of 0.7. The resulting clusters were visualized by t-distributed stochastic neighbor embedding (tSNE) and identified using the meta information files from TISCH database. Subsequently, we extracted the data for ECs from the Seurat object and repeated the aforementioned process with LogFC = 0.5 and min.pct = 0.35 to identify markers for different clusters.

### Cell culture and conditioned medium collection

As EC cells are difficult to extract from OS tissues, we selected the Human umbilical vein endothelial cells (HUVECs) as an *in vitro* model, based on previous references. The HUVECs were cultured in an endothelial growth medium supplemented with 10% fetal bovine serum and penicillin-streptomycin, as well as all the supplied endothelial growth medium supplements (Procel, Wuhan, China). For the preparation of endothelial cell-conditioned medium (ECs-CM), 1 × 10^6^ cells were seeded onto T25 culture flasks and incubated for 48 hours. The medium was then replaced with 5mL of serum-free DMEM/F12 (keyGEN, China) and further incubated for 24 h. The resulting CM was collected, filtered, and stored at −80°C. The human OS cell lines U2-OS and 143B (Procel, Wuhan, China) were cultured in DMEM/F12 supplemented with 10% FBS. All cells were cultured at 37°C in a humidified atmosphere of 95% air/5% CO_2_.

### Lentiviral transfection

LV-TYROBP and LV-NC were purchased from Sangon Biotech (Shanghai, China). HUVECs cells stably expressing TYROBP were established by transfecting the lentivirus.

### Enzyme-linked immunosorbent assay

Cell culture supernatants from normal HUVECs and TYROBP-positive HUVECs were collected to assess TWEAK secretion levels using a specific TWEAK ELISA kit (R&D Systems Inc., Minneapolis, MN). The color intensity of the wells was measured using a microplate reader (BioTek, Winooski, VT) at 450 nm after color development was stopped.

### Migration assay and Cell Counting Kit-8

We utilized Transwell chambers (8-µm pores) in a 24-well plate (Corning, MA, USA) to evaluate the migration of U2-OS and 143B. In the upper chamber, serum-free F12/DMEM containing tumor cells was added, while the lower chamber contained ECs-CM supplemented with 20% FBS. After culturing for 24 h, the migrated cells were fixed with 4% paraformaldehyde, stained with crystal violet, and enumerated using bright-field microscopy. The proliferation of U2-OS and 143B was assessed using a 96-well plate, with 2000 cells per well, and various concentrations of ECs-CM derived from normal HUVECs and TYROBP-positive HUVECs. The plate was incubated for two hours at 37°C, and the Cell Counting Kit-8 (CCK-8) reagent (Dojindo, Japan) was added before measuring absorbance at 450 nm. Assays were performed every 24 h.

### RNA extraction and real-time polymerase chain reaction (PCR) assay

RNA isolation was performed using the FastPure Cell/Tissue Total RNA Isolation Kit V2 (Vazyme, Nanjing, China) according to the manufacturer’s protocol. Complementary DNA (cDNA) was synthesized from the isolated RNA using the HiScript III All-in-one RT SuperMix Perfect for qPCR Kit (Vazyme, Nanjing, China). Real-time PCR was carried out on the synthesized cDNA using the Taq Pro Universal SYBR qPCR Master Mix Kit (Vazyme, Nanjing, China) in an ABI7500 real-time PCR system (Applied Biosystems). The primer sequence was from the previous references (15).

### Gene regulatory network analysis and pseudotime analysis

In order to identify the gene regulatory network of each cluster of ECs, we employed the single-cell regulatory network inference and clustering (SCENIC) approach using the SCENIC package (v1.3.1). The method infers potential transcription factor targets based on the expression data and evaluates the activity of regulons in individual cells. The differentially activated regulons of each cluster of ECs were identified by constructing a heatmap. Pseudotime analysis was conducted using the Monocle package (v2.22.0). Single-cell trajectories were calculated using the functions “order_cells” based on ECs cluster from Seurat.

### Cell–cell communication analysis

Cell-Cell Communication (CCC) Analysis serves as an essential reference database for establishing ligand-receptor pairs and secretory signaling pathways via the CellChat package (v1.1.3). The principal focus of CCC is to explore the relationship between malignant cells and ECs. To this end, the analytical suite, including “netAnalysis signalingRole scatter,” “netAnalysis signalingRole heatmap,” and “netVisual circle,” was utilized to gain valuable insight into the complex interplay between these cells.

### Gene set variation analysis and metabolic analysis

Gene set variation analysis (GSVA) (16) was executed utilizing the GSVA package (v1.40.1). Specifically, in the single-cell RNA sequencing dataset, the pathway activation was calculated for each cluster using “hallmark” gene sets. In addition, in the bulk RNA data, the score for each sample was evaluated using “KEGG.v7.4” gene sets, and a list of 29 immune cell-associated genes previously reported in the references was also considered. Metabolic analysis was executed utilizing the scMetabolism package (v0.2.1). The package was pre-populated with a human metabolic gene set, including 85 KEGG pathways and 82 REACTOME entries, based on the mean of the above gene sets values for metabolic activity analysis. These approaches provide a comprehensive and in-depth understanding of the gene expression pattern in these datasets.

### Pre-processing of the bulk transcriptome datasets

We utilized RNA-seq data from the TARGET database, including 88 OS samples, with non-coding RNAs excluded for downstream analysis. To ensure robustness of our modeling, we finally included 84 osteosarcoma samples without duplicate sequencing. To complement our findings, we also obtained the GSE21257 dataset from the GEO database, with samples screened using the same inclusion and exclusion criteria. An external validation cohort of 53 OS patients was included in our study. Finally, the specific endothelial cell cluster’s score of the bulk transcriptome sample was calculated using ssGSEA algorithm based on the marker genes derived from the scRNA-seq data. Overall patients were divided into high-score and low-score groups based on the cut-off of endothelial cell scores in survival analysis.

### ESTIMATE algorithm

We employed the ESTIMATE package (v1.0.13) to estimate stromal score and immune score of tumor samples based on their expression data. Furthermore, differential comparisons were conducted based on specific epithelial cells score.

### Construction and validation of the ECs-derived risk score

We used the limma package (v3.50.3) to calculate the differentially expressed genes (DEGs) between different epithelial cell score in the TARGET cohort. An adjusted p-value <0.05, and an absolute value of logFC greater than 1 were used as thresholds for selecting DEGs. We conducted a series of analyses to identify genes with prognostic value for our study. Initially, we performed univariate Cox regression analysis on the previously identified genes (p-value <0.05). Next, we applied a least absolute shrinkage and selection operator (LASSO) model to eliminate genes that were not useful for prognosis. We then constructed risk score formulas by integrating gene expression values weighted by their LASSO-Cox coefficients, as recommended in previous references’s pipline (17, 18). Finally, we assessed the independent prognostic value of the risk score in our dataset using multivariate Cox regression analysis. Additionally, we employed time-dependent subject operating characteristic (ROC) curves to compare the predictive accuracy of the risk score in different survival time. Finally, all patients were divided into high and low risk groups according to the cut-off value of risk score. For nomogram, based on our previous references, we utilized the regplot package (v1.1) to develop a nomogram that combined risk scores with significant clinical indicators identified by multivariate Cox regression analysis (19). To validate the prognostic predictive ability of the newly generated scores, we employed several packages, including survminer (v3.3-1), timeROC (v0.4), rms (v6.3-0), and rmda (v1.6). These packages were instrumental in assessing the performance of the scores and determining their usefulness in predicting outcomes.

### Drug sensitivity

The compDrugsen function of MOVICS package (v0.99.17) was used to compare the IC50 of drug of different groups by constructing ridge regression model.

### Statistical analysis

We conducted statistical analysis using R software (v4.1.2). For most cases, the significance of differences was tested using the Wilcoxon rank-sum test. The level of statistical significance was set at a p-value < 0.05, denoted by *p < 0.05, **p < 0.01, or ***p <0.001, while results without significant differences were marked as ns (not significant). We also performed overall survival analysis using the log-rank test.Results

### TYROBP-positive endothelial cells may be the initiating cells

**Figure 1:**
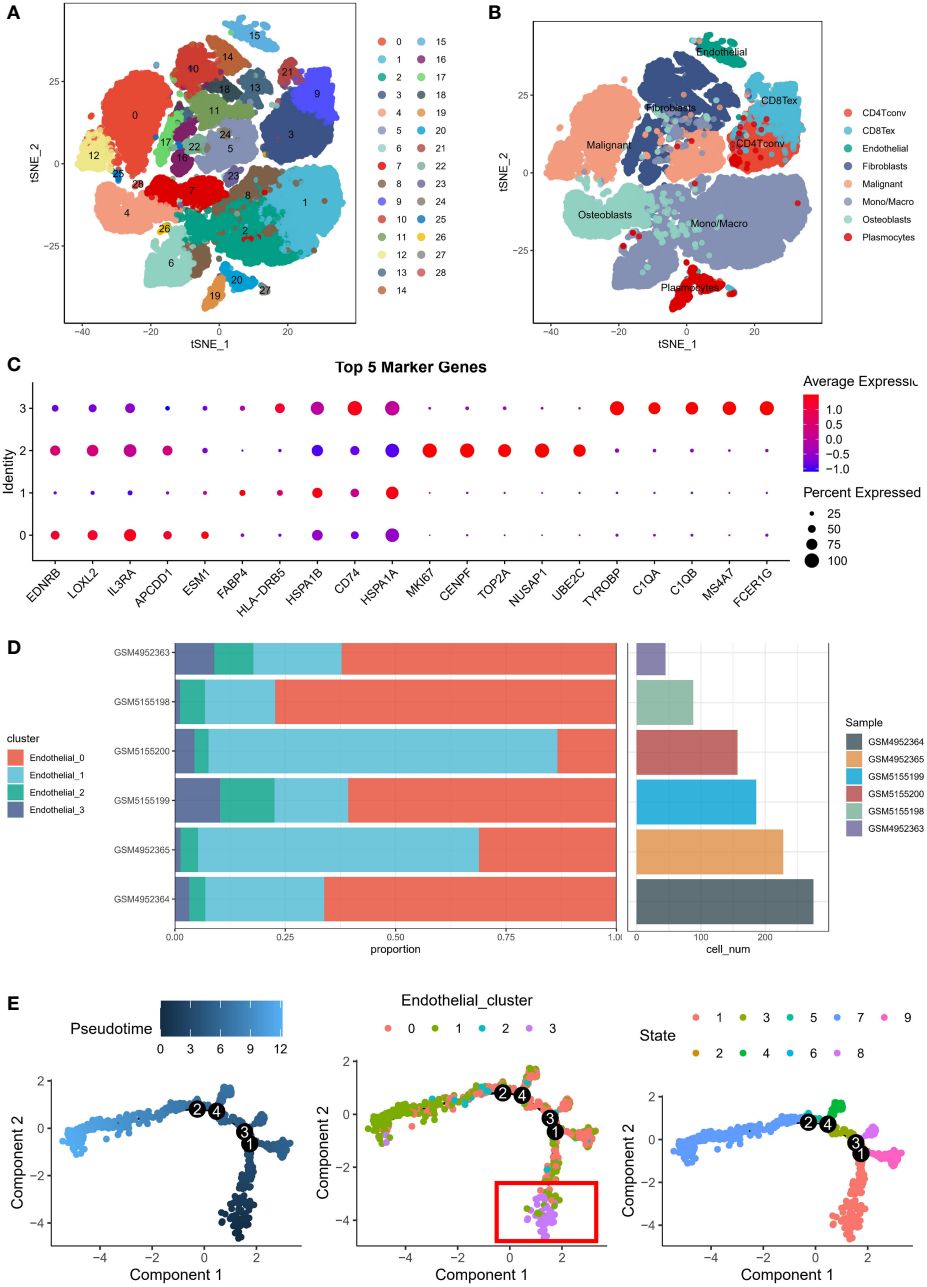
Endothelial cell atlas of osteosarcoma. **(A)** Clustering analysis of scRNA data from 6 osteosarcoma patients, which generated 29 subgroups. **(B)** Classification of cells into eight different cell types: plasma cells, conventional CD4+ T cells (CD4Tconv), exhausted CD8+ T cells (CD8Tex), endothelial cells, epithelial cells, fibroblasts, monocytes/macrophages (mono/macro), and osteoblasts. **(C)** Expression of highly expressed genes in four endothelial cell clusters: EDNBR (endothelial_0), FABP4 (endothelial_1), MKI67 (endothelial_2), and TYROBP (endothelial_3). **(D)** Percentage of different endothelial cells in six patients, with endothelial_0 and endothelial_1 being predominant in different patients. **(E)** Pseudotime analysis of endothelial cells, showing four nodes in the trajectory in two-dimensional space, with TYROBP-positive endothelial cells (endothelial_3) being the initiating cells.

We obtained the GSE162454 dataset from the TISCH database, which had been batch-corrected to minimize variations among samples. The dataset comprised of scRNA data from 6 OS patients, and these cells were clustered to yield 29 subgroups (**Figure 1A**). Using the annotation information provided in the TISCH database, the cells were classified into eight different cell types, namely, plasma cells, conventional CD4^+^ T cells (CD4Tconv), exhausted CD8^+^ T cells (CD8Tex), ECs, epithelial cells, fibroblasts, monocytes/macrophages (mono/macro), and osteoblasts (**Figure 1B**). As ECs are known to promote the progression of malignant cells (10), the original data was extracted from the endothelial cell clusters and re-clustered to construct an endothelial cell atlas, and the final ECs were divided into four clusters. The highly expressed genes in each cluster were identified as EDNBR (endothelial_0), FABP4 (endothelial_1), MKI67 (endothelial_2), and TYROBP (endothelial_3), as depicted in **Figure 1C**. To explore the specificity of different ECs, we determined the percentage of different ECs in six patients. For instance, endothelial_0 accounted for 62.2% in GSM4952363, endothelial_0 accounted for 66.2% in GSM4952364, endothelial_1 accounted for 63.6% in patient GSM4952365, endothelial_0 accounted for 77.3% in patient GSM5155198, endothelial_0 accounted for 60.1% in patient GSM5155199, and endothelial_1 accounted for 78.9% in patient GSM5155200 (**Figure 1D**). These results indicated that endothelial_0 and endothelial_1 was predominant in different patients, and there was heterogeneity in the distribution of other ECs among patients. To investigate the origin of differentiation of ECs, pseudotime analysis was performed, and interestingly, four nodes were identified in the trajectory in two-dimensional space after sorting the cells, with endothelial_3 being the initiating cells at the beginning (**Figure 1E**). Therefore, we speculate that TYROBP-positive ECs (endothelial_3) may play a crucial role in initiating the differentiation of ECs.

### TYROBP-positive endothelial cells generate the strongest crosstalk with malignant cells

**Figure 2:**
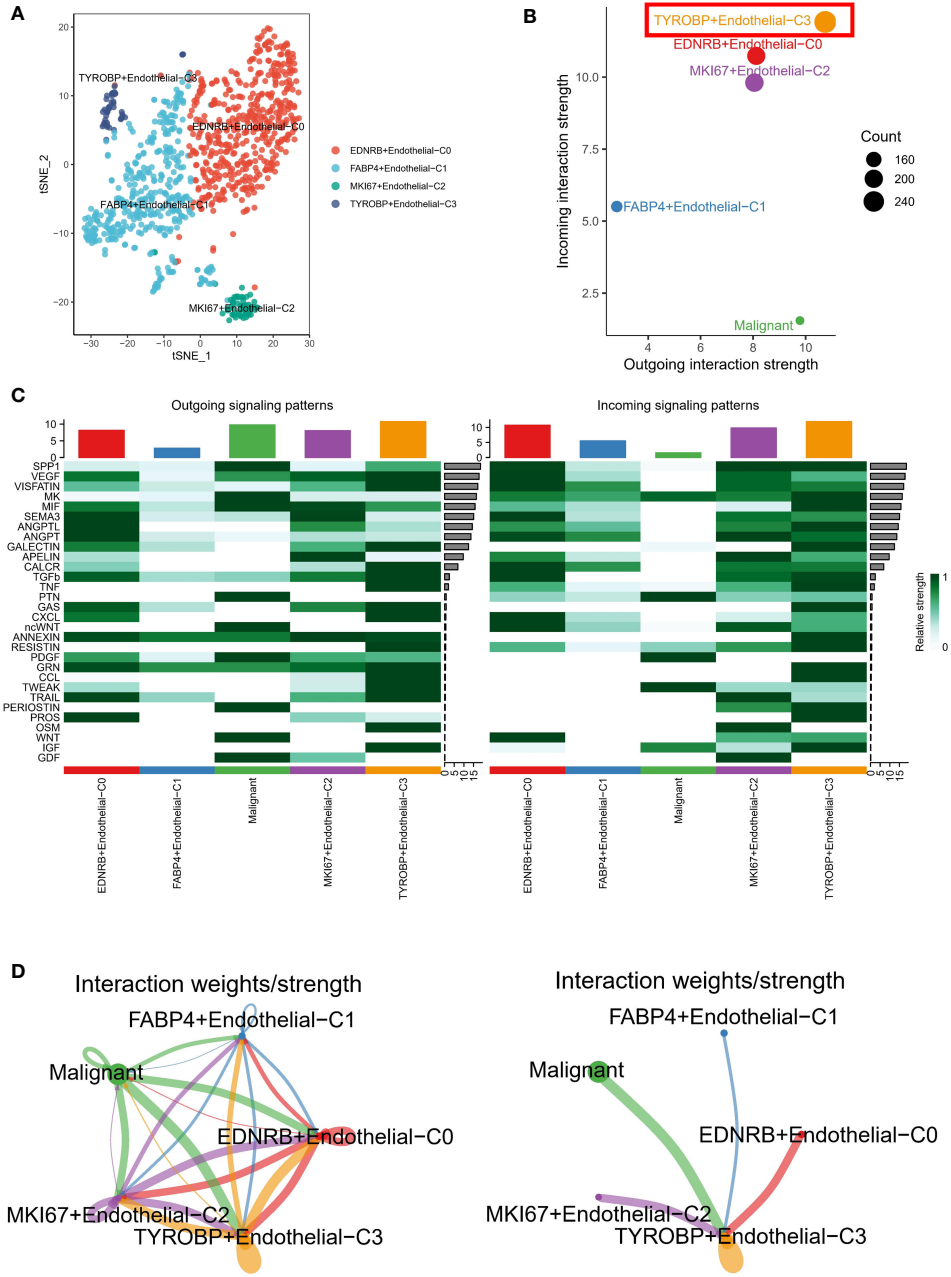
Analysis of endothelial cell subsets and interactions with malignant cells. **(A)** Tsne plot showing the four renamed subclusters of endothelial cells based on highly expressed markers. **(B)** TYROBP-positive ECs exhibit the highest incoming and outgoing signal strength, suggesting a critical role in communication between endothelial cell clusters and malignant cells. **(C)** Heatmap analysis demonstrates significant activation of key efferent signals, such as VEGF and CCL, in different cell types. **(D)** Network analysis reveals major interactions between malignant cells and TYROBP-positive ECs, MKI67-positive ECs, and EDNRB-positive ECs, as well as interconnections between different ECs.

We have re-annotated and visualized ECs using highly expressed markers of different subsets of ECs. Our analysis identified four renamed subclusters of endothelial cells, namely TYROBP-positive ECs, EDNRB-positive ECs, FABP4-positive ECs, and MKI67-positive ECs, as shown in the t-sne plot in **Figure 2A**. Interestingly, TYROBP-positive ECs displayed the highest incoming and outgoing signal strength, indicating that they may play a critical role in the communication between endothelial cell clusters and malignant cells (**Figure 2B**). We further examined the strength of secretory signals in different cell types using heatmaps, and our results demonstrated significant activation of key efferent signals such as VEGF and CCL (**Figure 2C**). Moreover, we used a network to calculate the weight and strength ratio of the interactions, and our findings revealed major interactions between malignant cells and TYROBP-positive ECs, MKI67-positive ECs, and EDNRB-positive ECs, as well as interconnections between different ECs (**Figure 2D**).

### Overexpression of TYROBP results in increased TWEAK production by endothelial cells

**Figure 3:**
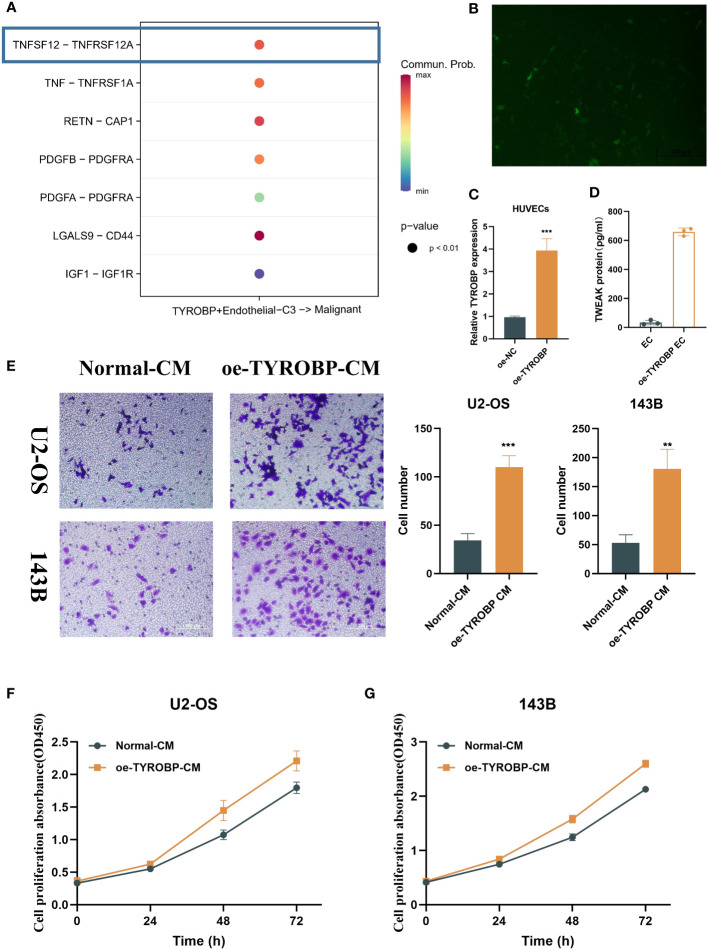
TYROBP-positive endothelial cells-derived TWEAK promote proliferation and migration of osteosarcoma. **(A)** Potential mechanism of interaction between TYROBP-positive ECs and malignant cells through the secretion of chemokines, including TNFSF12, TNF, RETN, and PDGFB, among others. **(B)** Fluorescence image 48h after transfection. **(C)** Relative mRNA expression of TYROBP in HUVECs cell lines after TYROBP overexpression. **(D)** TWEAK concentration in different conditioned medium. **(E)** Migration images of OS cell lines (U2-OS and 143B) treated with different conditioned medium. **(F)** The change of proliferative ability of U2-OS cell line under different conditioned medium. **(G)** The change of proliferative ability of 143B cell line under different conditioned medium. **P < 0.01; ***P < 0.001.

We investigated the potential mechanism by which TYROBP-positive ECs may interact with malignant cells through the secretion of chemokines, such as TNFSF12, TNF, RETN, and PDGFB, among others (**Figure 3A**). It is worth noting that TNSF12 showed the strongest communication scores. TNFSF12, also known as TNF-related weak inducer of apoptosis (TWEAK), is a cytokine that has been implicated in a variety of physiological and pathological processes. TNFSF12 is expressed by a range of cell types, including endothelial cells, and has been shown to interact with its receptor, fibroblast growth factor-inducible 14 (Fn14), to activate downstream signaling pathways. To investigate the impact of TYROBP overexpression in endothelial cells, we attempted to transfect TYROBP into HUVECs cell lines. However, due to the inherent characteristics of HUVECs, we encountered suboptimal transfection efficiency (**Figure 3B**). Nevertheless, we successfully confirmed the presence of TYROBP-positive endothelial cells through the detection of TYROBP mRNA (**Figure 3C**), which was further validated by ELISA data showing a significant increase in TWEAK production in the conditioned medium (**Figure 3D**). Notably, the conditioned medium from TYROBP-positive endothelial cells induced significant changes in the proliferation and migration of osteosarcoma cell lines (**Figures 3E–G**). Mechanistically, our data suggest that the elevated expression of TWEAK and other proteins in the TYROBP-positive endothelial cell-conditioned medium promoted the malignant phenotype of OS cells.

### TYROBP-positive endothelial cells have a unique metabolic and immunological profile

**Figure 4:**
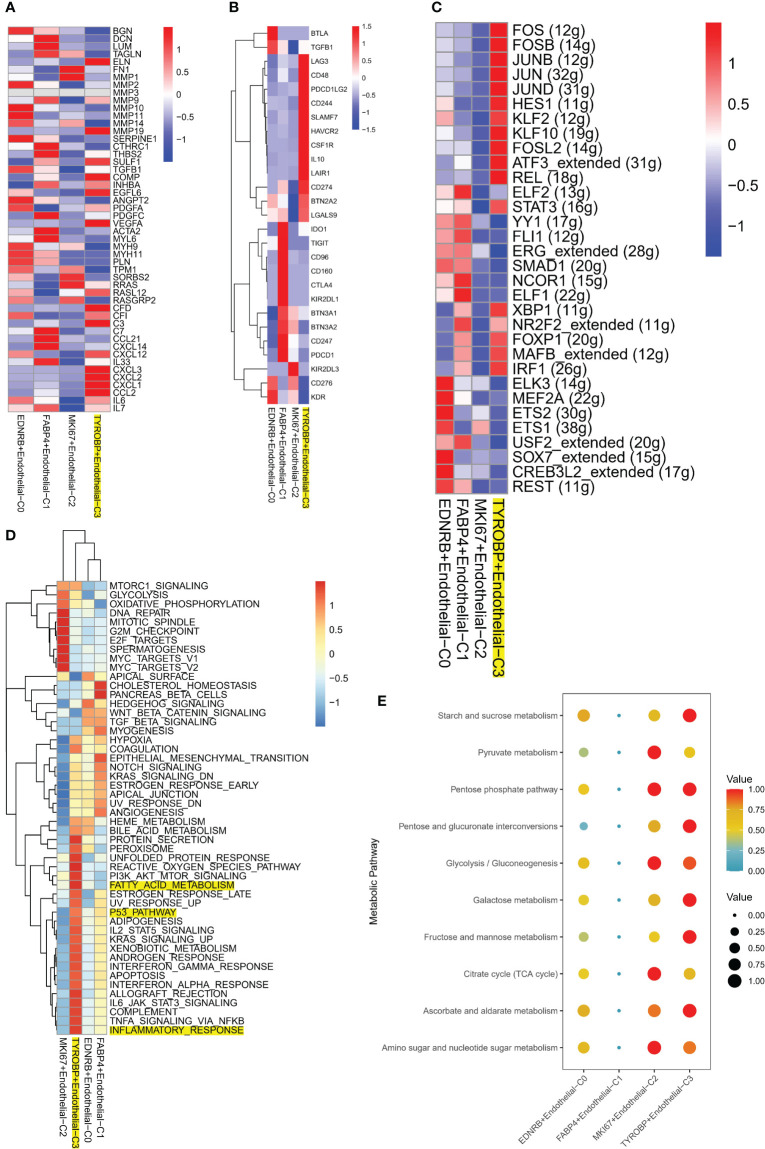
Molecular characteristics of TYROBP-positive endothelial cells in the tumor microenvironment. **(A)** TYROBP-positive endothelial cells showed significant expression of tumor microenvironment-related genes, including those involved in chemokine regulation such as CXCL12 and CXCL3. **(B)** Endothelial cell clusters exhibited differential expression of immune checkpoints, with TYROBP-positive endothelial cells expressing high levels of LAG3, CD48, PDCD1LG2, CD244, SLAMF7, and HAVCR2, while IDO1, TIGIT, CD95, CD160, and CTLA4 were highly expressed in FABP4-positive endothelial cells. **(C)** Analysis of gene regulatory networks revealed high activity of FOS, FOSB, HES1, and JUNB in TYROBP-positive endothelial cells, suggesting that these transcription factors may promote the differentiation of initiating cells to other isoforms. **(D)** Pathway analysis showed that TYROBP-positive endothelial cells were highly activated in fatty acid metabolic signaling pathways, inflammatory responses, and pro-oncogenic pathways, such as p53 and KRAS. **(E)** A heatmap of the top 10 activated metabolism-related pathways showed that FABP4-positive endothelial cells were in a metabolic silent state, while MKI67-positive endothelial cells were dominated by pyruvate metabolism. In contrast, TYROBP-positive endothelial cells were primarily engaged in starch and sucrose metabolism, highlighting distinct metabolic pathway utilization between different endothelial cell clusters in the tumor microenvironment.

Notably, TYROBP-positive ECs exhibited significant expression of TME-related genes, including those regulating chemokine expression, such as CXCL12 and CXCL3, as demonstrated in **Figure 4A**. Additionally, differential expression of immune checkpoints (ICI) on ECs clusters was observed, with LAG3, CD48, PDCD1LG2, CD244, SLAMF7, and HAVCR2 being highly expressed in TYROBP-positive ECs, while IDO1, TIGIT, CD95, CD160, and CTLA4 were highly expressed in FABP4-positive ECs, as depicted in **Figure 4B**. Subsequently, we examined the gene regulatory network to elucidate the transcription factors (TFs) activity changes during the conversion process. Our findings revealed that TYROBP-positive ECs exhibited high activity of FOS, FOSB, HES1, and JUNB, indicating that these TFs may promote the differentiation of initiating cells (TYROBP-positive ECs) to other isoforms, as illustrated in **Figure 4C**. Pathway analysis demonstrated that TYROBP-positive ECs were highly activated in fatty acid metabolic signaling pathways, inflammatory responses, and pro-oncogenic pathways, such as p53 and KRAS, as presented in **Figure 4D**. Therefore, we further investigated the differences in metabolic pathways between ECs clusters and found that they were distinct. The heat map of the top 10 activated metabolism-related pathways showed that FABP4-positive ECs were in a metabolic silent state, while MKI67-positive ECs were dominated by pyruvate metabolism, and TYROBP-positive ECs were primarily engaged in starch and sucrose metabolism (**Figure 4E**).

### High enrichment of TYROBP-positive endothelial cells indicates a better prognosis

**Figure 5:**
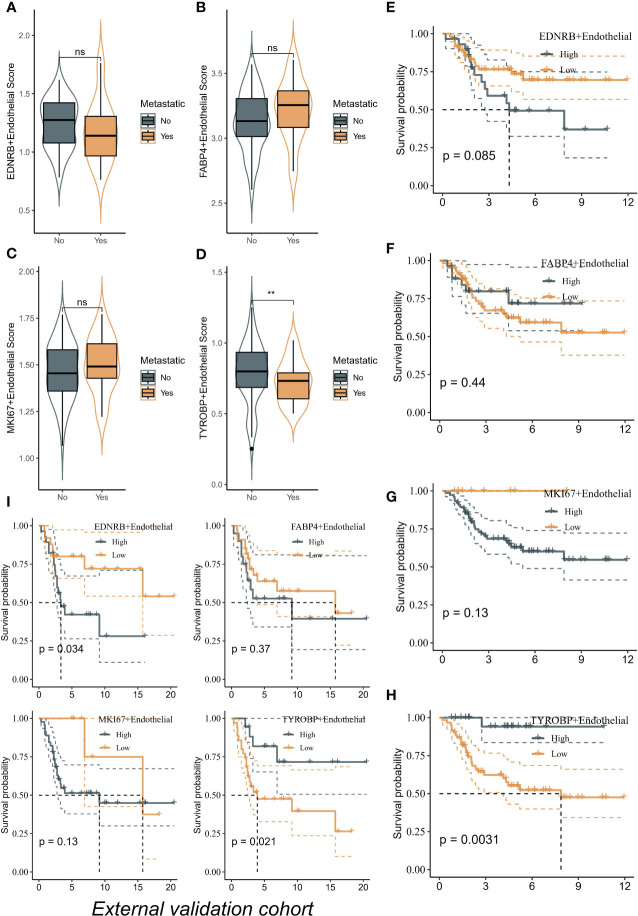
TYROBP-positive endothelial cells as potential suppressors of tumor metastasis and prognostic indicators in osteosarcoma. **(A)** The scores of EDNRB-positive ECs in metastatic samples and no-metastatic samples. **(B)** The scores of FABP4-positive ECs in metastatic samples and no-metastatic samples. **(C)** The scores of MKI67-positive ECs in metastatic samples and no-metastatic samples. **(D)** The scores of TYROBP-positive ECs in metastatic samples and no-metastatic samples. TYROBP-positive endothelial cells were found to be downregulated in metastatic samples in the TARGET-OS cohort. Kaplan-Meier plots of the prognostic impact of different endothelial cell types on patient outcomes in the TARGET-OS cohort, including EDNRB-positive ECs **(E)**, FABP4-positive ECs **(F)**, and MKI67-positive ECs **(G)** and TYROBP-positive ECs **(H)**. **(I)** Kaplan-Meier plots of the prognostic impact of different endothelial cell types on patient outcomes in the GSE21257 cohort. **p < 0.01, ns (not significant).

We utilized the ssGSEA algorithm to assess different ECs scores per patient from bulk transcriptome data. Remarkably, only TYROBP-positive ECs were found to be downregulated in metastatic samples in the TARGET-OS cohort, indicating its potential role in inhibiting tumor metastasis (**Figures 5A–D**). Additionally, we evaluated the prognostic impact of different ECs on patient outcomes in the TARGET-OS cohort, and only TYROBP-positive ECs exhibited significant prognostic implications. Specifically, patients with low levels of enriched TYROBP-positive ECs had a poorer prognosis (**Figures 5E–H**). Notably, the prognostic power of TYROBP-positive ECs was consistently observed in the validation cohort GSE21257 (**Figure 5I**).

### High enrichment of TYROBP-positive endothelial cells represents the “hot” tumor state

**Figure 6:**
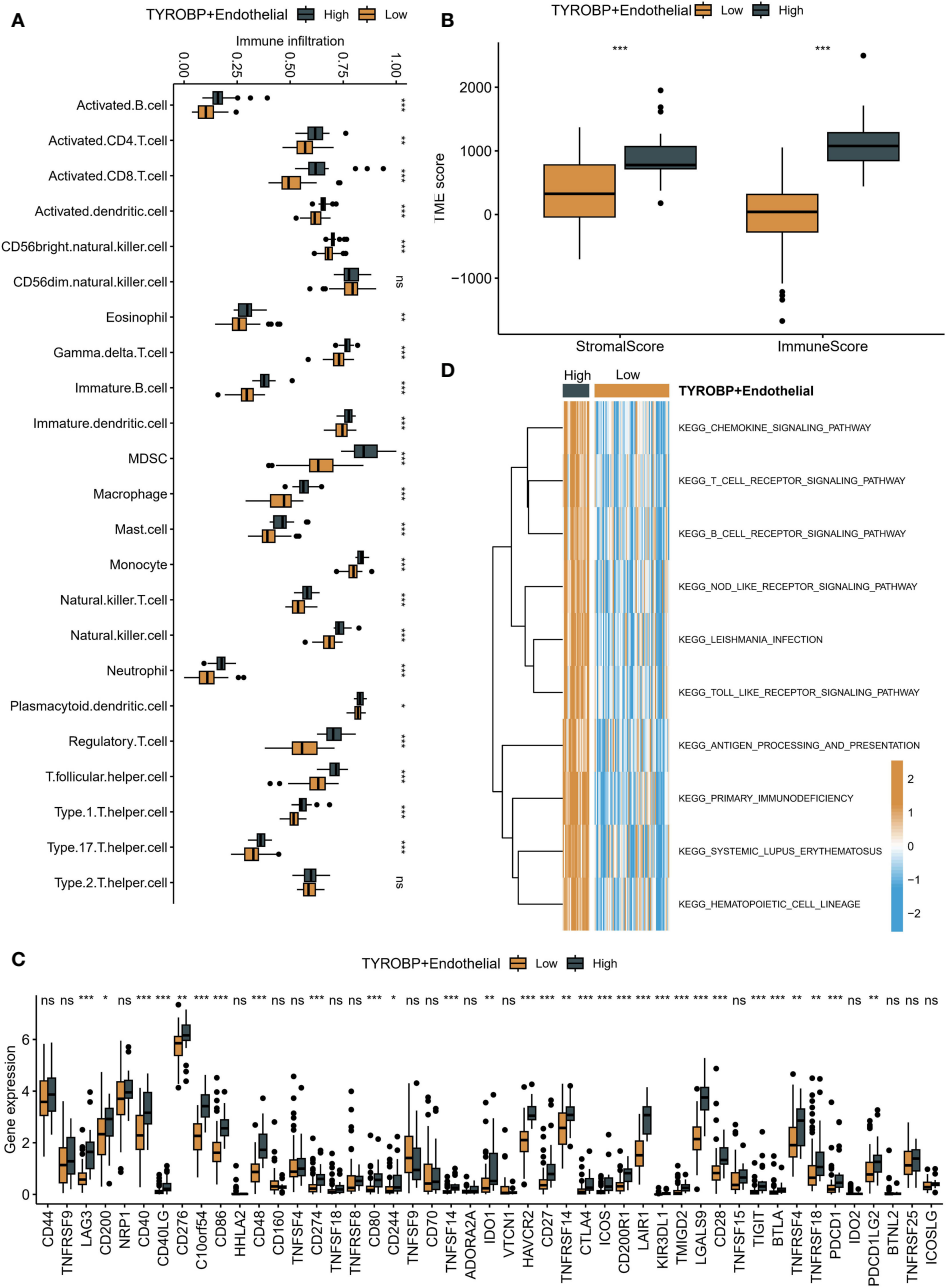
Immune microenvironment differences in patients with varying TYROBP-positive EC enrichment levels. **(A)** Patients with high enrichment of TYROBP-positive ECs had increased numbers of activated CD4+ T cells, CD8+ T cells, and natural killer cells. **(B)** Patients with highly enriched TYROBP-positive ECs also exhibited higher immune scores, indicative of a “hot” tumor state, as determined by the ESTIMATE algorithm. **(C)** Patients with highly enriched ECs had higher expression of classical immune checkpoint inhibitors (ICIs). **(D)** High enrichment of TYROBP-positive ECs was associated with significantly activated chemokine, T cell receptor, B cell receptor, and Nod-like receptor signaling pathways, indicating a “hot” tumor state. *P < 0.05; **P < 0.01; ***P < 0.001; ns represents no statistical significance.

Using the GSVA algorithm, we observed differences in the immune microenvironment among patients with varying enrichment levels of TYROBP-positive ECs. Interestingly, patients with high enrichment of TYROBP-positive ECs had greater numbers of activated CD4^+^ T cells, CD8^+^ T cells, and natural killer cells (**Figure 6A**). To ensure the robustness of our findings, we further used the ESTIMATE algorithm to determine that patients with highly enriched TYROBP-positive ECs also exhibited higher immune scores, indicative of a “hot” tumor state (**Figure 6B**). Additionally, we compared the mRNA expression levels of immune checkpoint inhibitors (ICIs) in patients with different EC enrichments and found that those with highly enriched ECs had higher expression of classical ICIs, such as CD276 and CD274 (**Figure 6C**). Finally, we evaluated the activation of KEGG pathways among different patients and found that high enrichment of TYROBP-positive ECs was associated with significantly activated chemokine, T cell receptor, B cell receptor, and Nod-like receptor signaling pathways, further supporting our conclusion that it corresponds to a “hot” tumor state. (**Figure 6D**).

### TYROBP-positive endothelial cell-derived risk signature can be used for prognostic prediction and medication guidance

**Figure 7:**
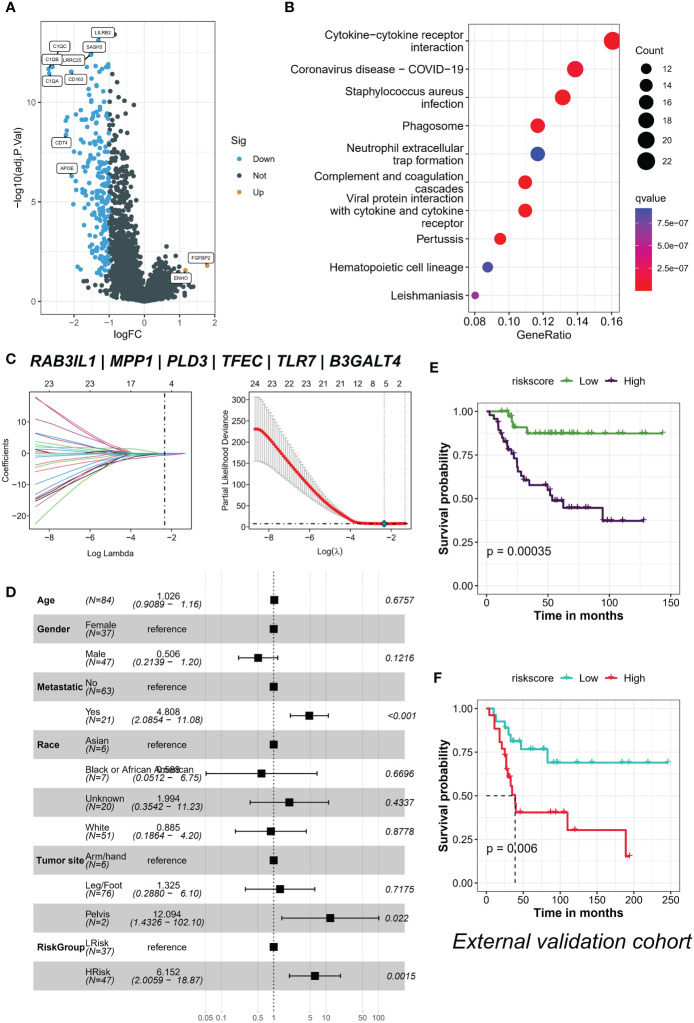
TYROBP-positive endothelial cell enrichment is associated with immunity and is an independent prognostic factor. **(A)** Volcano plot depicting the 213 differentially expressed genes (DEGs) identified between patients with varying levels of TYROBP-positive ECs enrichment. **(B)** KEGG enrichment analysis demonstrating that cytokine-cytokine receptor interaction is the most enriched pathway associated with TYROBP-positive ECs. **(C)** LASSO regression model selecting 6 TYROBP-positive endothelial cell-derived genes for risk score construction. **(D)** Multifactorial Cox regression analysis showing that risk grouping based on the constructed risk score is an independent prognostic factor, even after adjusting for clinicopathological variables. Kaplan-Meier curves depicting the survival of patients in different cohorts, such as TARGERT-OS cohort **(E)** and GSE21257 **(F)**, divided into high-risk and low-risk groups based on the constructed risk score.

**Figure 8:**
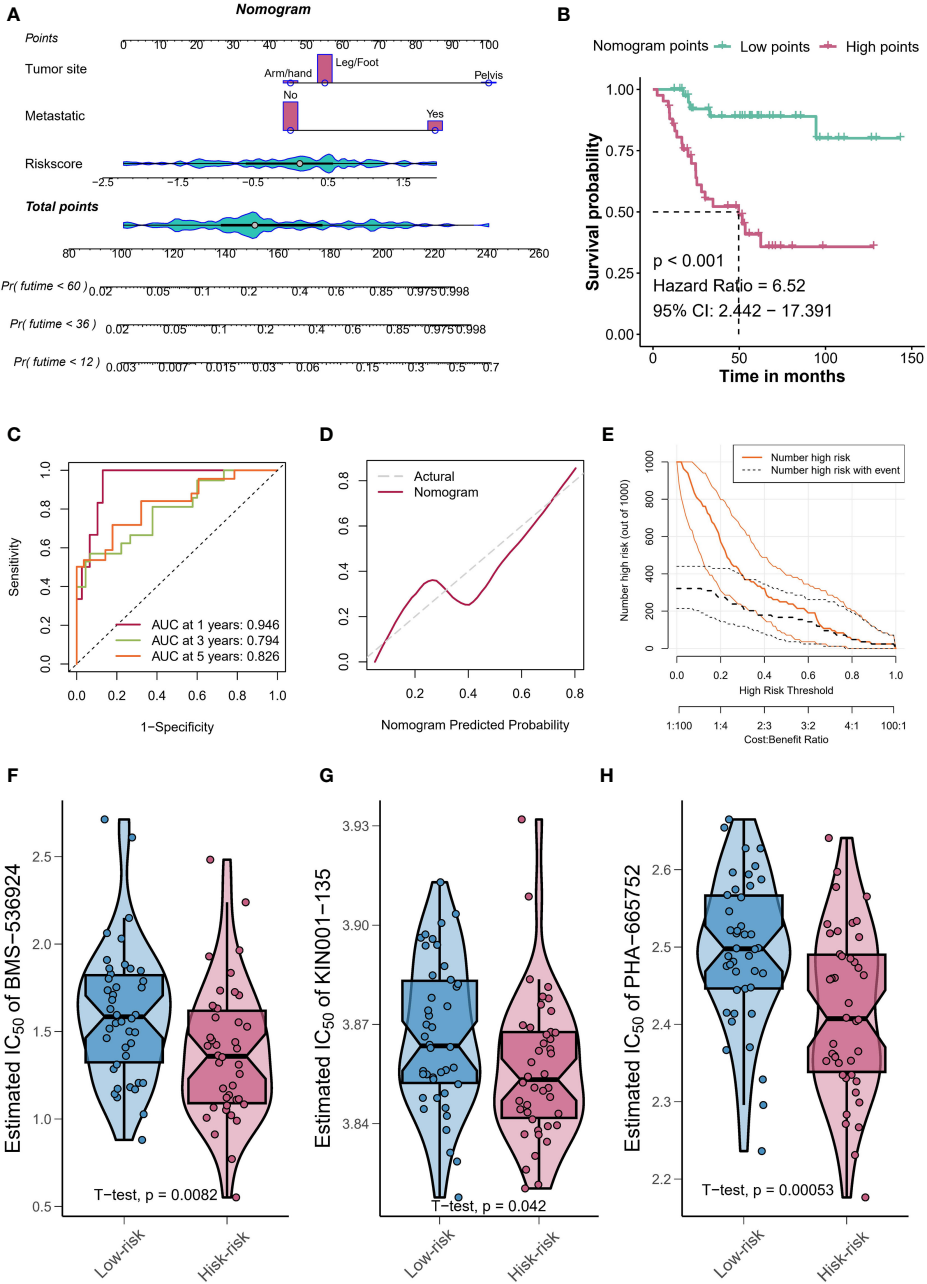
Nomograms and drug sensitivity analysis. **(A)** Nomograms based on clinical variables and risk scores demonstrate clinical benefit. **(B)** Kaplan-Meier curves depicting the survival of patients with different nomogram’s points. **(C)** The calibration curve shows the agreement between predicted and actual outcomes, further validating the clinical benefit of using the points-based classification scheme. **(D)** The clinical impact curve demonstrates the clinical benefit of using the points-based classification scheme. **(E)** The clinical impact curve demonstrates the clinical benefit of using the points-based classification scheme. Drug sensitivity analysis using BMS-536924 **(F)**, KIN001 **(G)**, and PHA-665752 **(H)** reveals greater sensitivity to these drugs in the high-risk group.

We identified 213 differential expression genes (DEGs) between patients with varying levels of TYROBP-positive EC enrichment (**Figure 7A**). Further KEGG enrichment analysis revealed a significant association between TYROBP-positive ECs and immunity, with cytokine-cytokine receptor interaction being the most enriched pathway (**Figure 7B**). Using LASSO-Cox model, we identified 35 overlapping prognostic genes from the validation cohort, and subsequently selected 6 TYROBP-positive endothelial cell-derived genes for risk score construction (**Figure 7C**). We divided patients into high-risk and low-risk groups using a cut-off value, and found that risk grouping was an independent prognostic factor, even after adjusting for clinicopathological variables (**Figure 7D**). The high-risk group had a better prognosis in different cohorts (**Figures 7E, F**). To aid clinical use, we developed nomograms based on meaningful clinical variables and risk scores, which demonstrated clinical benefit in ROC curve, calibration curve, and clinical impact curve analyses (**Figures 8A–E**). Additionally, we performed drug sensitivity analysis using three targeted drugs with therapeutic OS potential, including BMS-536924, KIN001, and PHA-665752. Our findings showed that the IC50 of the high-risk array was significantly lower than that of the low-risk array, indicating greater sensitivity to these drugs in the high-risk group (**Figures 8F–H**).

In summary, our findings suggest that TYROBP-positive EC enrichment plays a critical role in promoting immunity and can be used as an independent prognostic feature to predict patient outcomes. The development of visualized nomograms and drug sensitivity analysis further demonstrate the clinical benefit of using this classification scheme.

## Discussion

ECs play a critical role in the TME and have been shown to promote tumor progression by providing oxygen and nutrients to tumor cells, regulating immune cell infiltration, and mediating tumor cell intravasation and extravasation during metastasis (20). In this study, we utilized scRNA-seq and bulk transcriptome to investigate the heterogeneity of ECs in OS and identify potential initiating cells and their role in tumor progression.

TYROBP, also known as DAP12, is a human gene located on chromosome 19q13.1. It encodes a transmembrane adaptor protein that is mainly expressed in cells, and the protein product of TYROBP is involved in a variety of immune responses, including regulating the activation and function of natural killer cells and dendritic cells (21). It forms complexes with cell surface receptors, such as triggering receptor expressed on myeloid cells 1 (TREM1), which activate downstream signaling pathways (22). Mutations in TYROBP have been associated with various diseases, including Nasu-Hakola disease, which is a rare neurodegenerative disorder that affects the brain and bones (23). Additionally, TYROBP has been implicated in the pathogenesis of Alzheimer’s disease and other neurodegenerative diseases (24, 25). Overall, TYROBP plays a crucial role in immune system function and has implications for various diseases, particularly those affecting the brain and immune system. Our results revealed that TYROBP-positive ECs displayed the highest incoming and outgoing signal strength when communicating with malignant cells. We also found that TYROBP-positive ECs exhibited significant expression of TME-related genes, including those regulating chemokine expression, and had high activity of transcription factors FOS, FOSB, HES1, and JUNB. These findings suggest that TYROBP-positive ECs may play a crucial role in regulating immune cell infiltration and promoting tumor progression through their interactions with malignant cells. Moreover, our analysis identified differential expression of immune checkpoints (ICI) on ECs clusters. LAG3, CD48, PDCD1LG2, CD244, SLAMF7, and HAVCR2 were highly expressed in TYROBP-positive ECs, while IDO1, TIGIT, CD95, CD160, and CTLA4 were highly expressed in FABP4-positive ECs. This differential expression of ICIs suggests that TYROBP-positive ECs may play a unique role in immune evasion in the TME.

There have been successful precedents for converting “cold” tumors to “hot” tumors and improving the efficacy of immunotherapy. “Cold” tumors are characterized by a lack of T cell infiltration, whereas “hot” tumors are characterized by high levels of T cell infiltration and activity (26). Immunotherapy, such as immune checkpoint inhibitors, can be highly effective in treating “hot” tumors, but has limited efficacy in “cold” tumors. We evaluated the activation of KEGG pathways among different patients and found that high enrichment of TYROBP-positive ECs was associated with significantly activated chemokine, T cell receptor, B cell receptor, and Nod-like receptor signaling pathways, further supporting our conclusion that it corresponds to a “hot” tumor state.

Studies have shown that the TYROBP gene may play a role in the development and progression of certain cancers. For example, TYROBP expression has been found to be increased in several types of cancer, including breast cancer (27), lung cancer (28), and low-grade glioma (8). In breast cancer, higher levels of TYROBP expression have been associated with worse clinical outcomes, including shorter overall survival and disease-free survival (27). In low-grade glioma, TYROBP has been shown to promote tumor growth and metastasis through its interaction with other proteins involved in cell signaling pathways (8). Although the exact mechanisms by which TYROBP contributes to tumorigenesis are not yet fully understood, these findings suggest that TYROBP may be a potential target for cancer therapy and highlight the need for further research into its role in cancer development and progression.

Currently, numerous studies have investigated the relationship between ECs and osteosarcoma; however, a specific subtype of ECs has not been the primary focus of most of these reports. ECs promote osteosarcoma progression by secreting Von Willebrand factor (VWF) and activating NF-κB signaling, which contributes to epithelial-mesenchymal transition (EMT) and metastasis; OS cells in turn activate phospholipase D1 signaling to promote VWF release by ECs, resulting in further tumor deterioration (29). Kanako Minami and his colleagues found that Free fatty acid receptor 1 (FFA1) and FFA4, which regulate various malignant properties in cancer cells, may play a significant role in the modulation of cellular functions by ECs in OS, with higher expression levels of FFAR1 and FFAR4 genes in highly migratory MG63-CR7(F2) cells than in MG-63 cells, and knockdown of FFA1 or FFA4 inhibiting cell survival in the presence of cisplatin (30). Filomena de Nigris a suggests that Cyclin-dependent kinase 2 and 5 (Cdk2, Cdk5) are important mediators of neoangiogenesis in osteosarcoma, and a specific Yin Yang 1 (YY1) protein-dependent signal from tumor cells determines proliferation of human ECs, with Roscovitine inhibiting Cdk2 and Cdk5 activity, decreasing ECs proliferation and angiogenesis, and potentially serving as a pharmacologically accessible target for both antiangiogenic and antitumor therapy (31). However, we concluded that TYROBP-positive ECs may be the initiating cells and target specific subgroups of ECs that may benefit patients with OS.

It is worth noting that our study has several limitations. About vitro experiment, the main limitation is the suboptimal transfection efficiency of HUVECs cell lines, which may have resulted in incomplete TYROBP overexpression. Additionally, the study did not investigate the specific mechanisms underlying the changes in osteosarcoma cell proliferation and migration induced by the TYROBP-positive endothelial cell-conditioned medium. Finally, while the data suggests a role for TWEAK and other proteins in promoting the malignant phenotype of osteosarcoma cells, the study did not provide conclusive evidence regarding the specific proteins or pathways involved. Moreover, our sample size is relatively small and that it may limit the generalizability of our findings. Our analysis was based on a single scRNA-seq dataset from the TISCH database, which may not fully represent the heterogeneity of ECs in OS. Further validation with additional datasets is necessary to confirm our findings. Second, we focused on the role of TYROBP-positive ECs in tumor progression, but other EC clusters may also play important roles in the TME. Finally, our study was based on computational analysis and further experimental validation is necessary to confirm our findings.

## Conclusions

In conclusion, our study identified TYROBP-positive ECs as the initiating cells of differentiation in ECs clusters in OS, and suggested that they may play a crucial role in promoting tumor progression through their interactions with malignant cells and regulating immune cell infiltration in the TME. In the future, targeting TYROBP-positive ECs maybe represent a promising approach for the treatment of OS and holds great potential for improving patient outcomes.

## Data availability statement

The original contributions presented in the study are included in the article/supplementary material. Further inquiries can be directed to the corresponding authors.

## Author contributions

Z-QW conceived and designed the study. SD drafted the article. Y-CY revised the article critically. All authors contributed to the article and approved the submitted version.

## References

[1] Eaton BR Schwarz R Vatner R Yeh B Claude L Indelicato DJ. Osteosarcoma. Pediatr Blood Cancer (2021) 68 Suppl 2:e28352. doi: 10.1002/pbc.28352 32779875

[2] Cole S Gianferante DM Zhu B Mirabello L. Osteosarcoma: a surveillance, epidemiology, and end results program-based analysis from 1975 to 2017. Cancer (2022) 128:2107–18. doi: 10.1002/cncr.34163 PMC11647566 35226758

[3] Kong C Hansen MF. Biomarkers in osteosarcoma. Expert Opin Med Diagn (2009) 3:13–23. doi: 10.1517/17530050802608496 20574545 PMC2889491

[4] Dana PM Sadoughi F Asemi Z Yousefi B. Molecular signaling pathways as potential therapeutic targets in osteosarcoma. Curr Med Chem (2022) 29:4436–44. doi: 10.2174/0929867329666220209110009 35139778

[5] Harrison DJ Geller DS Gill JD Lewis VO Gorlick R. Current and future therapeutic approaches for osteosarcoma. Expert Rev Anticancer Ther (2018) 18:39–50. doi: 10.1080/14737140.2018.1413939 29210294

[6] Rickel K Fang F Tao J. Molecular genetics of osteosarcoma. Bone (2017) 102:69–79. doi: 10.1016/j.bone.2016.10.017 27760307 PMC5393957

[7] Amersfoort J Eelen G Carmeliet P. Immunomodulation by endothelial cells - partnering up with the immune system? Nat Rev Immunol (2022) 22:576–88. doi: 10.1038/s41577-022-00694-4 PMC8920067 35288707

[8] Lu J Peng Y Huang R Feng Z Fan Y Wang H. Elevated TYROBP expression predicts poor prognosis and high tumor immune infiltration in patients with low-grade glioma. BMC Cancer (2021) 21:723. doi: 10.1186/s12885-021-08456-6 34162355 PMC8220692

[9] Huang Q Liang X Ren T Huang Y Zhang H Yu Y. The role of tumor-associated macrophages in osteosarcoma progression - therapeutic implications. Cell Oncol (Dordr) (2021) 44:525–39. doi: 10.1007/s13402-021-00598-w PMC12980758 33788151

[10] Maishi N Hida K. Tumor endothelial cells accelerate tumor metastasis. Cancer Sci (2017) 108:1921–6. doi: 10.1111/cas.13336 PMC5623747 28763139

[11] Liu S Qin T Liu Z Wang J Jia Y Feng Y. Anlotinib alters tumor immune microenvironment by downregulating PD-L1 expression on vascular endothelial cells. Cell Death Dis (2020) 11:309. doi: 10.1038/s41419-020-2511-3 32366856 PMC7198575

[12] Xia Y Wang WC Shen WH Xu K Hu YY Han GH. Thalidomide suppresses angiogenesis and immune evasion via lncRNA FGD5-AS1/miR-454-3p/ZEB1 axis-mediated VEGFA expression and PD-1/PD-L1 checkpoint in NSCLC. Chem Biol Interact (2021) 349:109652. doi: 10.1016/j.cbi.2021.109652 34520751

[13] Nam AS Chaligne R Landau DA. Integrating genetic and non-genetic determinants of cancer evolution by single-cell multi-omics. Nat Rev Genet (2021) 22:3–18. doi: 10.1038/s41576-020-0265-5 32807900 PMC8450921

[14] Sun D Wang J Han Y Dong X Ge J Zheng R. TISCH: a comprehensive web resource enabling interactive single-cell transcriptome visualization of tumor microenvironment. Nucleic Acids Res (2021) 49:D1420–30. doi: 10.1093/nar/gkaa1020 PMC7778907 33179754

[15] Li S Zhang Q Huang Z Tao W Zeng C Yan L. Comprehensive analysis of immunocyte infiltration and the key genes associated with intraplaque hemorrhage in carotid atherosclerotic plaques. Int Immunopharmacol (2022) 106:108633. doi: 10.1016/j.intimp.2022.108633 35183915

[16] Hanzelmann S Castelo R Guinney J. GSVA: gene set variation analysis for microarray and RNA-seq data. BMC Bioinf (2013) 14:7. doi: 10.1186/1471-2105-14-7 PMC3618321 23323831

[17] Feng S Xu Y Dai Z Yin H Zhang K Shen Y. Integrative analysis from multicenter studies identifies a WGCNA-derived cancer-associated fibroblast signature for ovarian cancer. Front Immunol (2022) 13:951582. doi: 10.3389/fimmu.2022.951582 35874760 PMC9304893

[18] Feng S Yin H Zhang K Shan M Ji X Luo S. Integrated clinical characteristics and omics analysis identifies a ferroptosis and iron-metabolism-related lncRNA signature for predicting prognosis and therapeutic responses in ovarian cancer. J Ovarian Res (2022) 15:10. doi: 10.1186/s13048-022-00944-y 35057848 PMC8772079

[19] Zhang K Feng S Ge Y Ding B Shen Y. A nomogram based on SEER database for predicting prognosis in patients with mucinous ovarian cancer: a real-world study. Int J Womens Health (2022) 14:931–43. doi: 10.2147/IJWH.S372328 PMC9341457 35924098

[20] Nagl L Horvath L Pircher A Wolf D. Tumor endothelial cells (TECs) as potential immune directors of the tumor microenvironment - new findings and future perspectives. Front Cell Dev Biol (2020) 8:766. doi: 10.3389/fcell.2020.00766 32974337 PMC7466447

[21] Tomasello E Vivier E. KARAP/DAP12/TYROBP: three names and a multiplicity of biological functions. Eur J Immunol (2005) 35:1670–7. doi: 10.1002/eji.200425932 15884055

[22] Chen B Zhou M Zhang H Wang C Hu X Wang B. TREM1/Dap12-based CAR-T cells show potent antitumor activity. Immunotherapy (2019) 11:1043–55. doi: 10.2217/imt-2019-0017 31268375

[23] Satoh JI Yanaizu M Tosaki Y Sakai K Kino Y. Targeted sequencing approach to identify genetic mutations in nasu-hakola disease. Intractable Rare Dis Res (2016) 5:269–74. doi: 10.5582/irdr.2016.01064 PMC5116862 27904822

[24] Ma J Jiang T Tan L Yu JT. TYROBP in alzheimer’s disease. Mol Neurobiol (2015) 51:820–6. doi: 10.1007/s12035-014-8811-9 25052481

[25] Haure-Mirande JV Audrain M Ehrlich ME Gandy S. Microglial TYROBP/DAP12 in alzheimer’s disease: transduction of physiological and pathological signals across TREM2. Mol Neurodegener (2022) 17:55. doi: 10.1186/s13024-022-00552-w 36002854 PMC9404585

[26] Zhang J Huang D Saw PE Song E. Turning cold tumors hot: from molecular mechanisms to clinical applications. Trends Immunol (2022) 43:523–45. doi: 10.1016/j.it.2022.04.010 35624021

[27] Shabo I Olsson H Stal O Svanvik J. Breast cancer expression of DAP12 is associated with skeletal and liver metastases and poor survival. Clin Breast Cancer (2013) 13:371–7. doi: 10.1016/j.clbc.2013.05.003 23810293

[28] Kettunen E Anttila S Seppanen JK Karjalainen A Edgren H Lindstrom I. Differentially expressed genes in nonsmall cell lung cancer: expression profiling of cancer-related genes in squamous cell lung cancer. Cancer Genet Cytogenet (2004) 149:98–106. doi: 10.1016/S0165-4608(03)00300-5 15036884

[29] Ling J Sun Y Pan J Wang H Ma Z Yin J. Feedback modulation of endothelial cells promotes epithelial-mesenchymal transition and metastasis of osteosarcoma cells by Von willebrand factor release. J Cell Biochem (2019) 120:15971–9. doi: 10.1002/jcb.28875 31099074

[30] Minami K Ueda N Ishimoto K Tsujiuchi T. Regulation of cell survival through free fatty acid receptor 1 (FFA1) and FFA4 induced by endothelial cells in osteosarcoma cells. J Recept Signal Transduct Res (2020) 40:181–6. doi: 10.1080/10799893.2020.1725047 32026734

[31] de Nigris F Mancini FP Schiano C Infante T Zullo A Minucci PB. Osteosarcoma cells induce endothelial cell proliferation during neo-angiogenesis. J Cell Physiol (2013) 228:846–52. doi: 10.1002/jcp.24234 23042366

